# Identification of metabolism-related key genes as potential biomarkers for pathogenesis of immune thrombocytopenia

**DOI:** 10.1038/s41598-024-59493-7

**Published:** 2024-04-19

**Authors:** Xiangmei Xu, Jiamin Zhang, Hongyun Xing, Liying Han, Xiaoming Li, Pengqiang Wu, Jirui Tang, Li Jing, Jie Luo, Jing Luo, Lin Liu

**Affiliations:** 1 Department of Hematology, The First Affiliated Hospital of Chongqing Medical University, No. 1 Youyi Road, Yuzhong District, Chongqing, 400016 People’s Republic of China; 2 Department of Oncology and Hematology, The Affiliated Traditional Chinese Medicine Hospital, Southwest Medical University, Luzhou, China; 3 Department of Hematology, The Affiliated Hospital, Southwest Medical University, Luzhou, China

**Keywords:** Computational biology and bioinformatics, Biomarkers, Haematological diseases

## Abstract

Immune thrombocytopenia (ITP), an acquired autoimmune disease, is characterized by immune-mediated platelet destruction. A biomarker is a biological entity that contributes to disease pathogenesis and reflects disease activity. Metabolic alterations are reported to be associated with the occurrence of various diseases. As metabolic biomarkers for ITP have not been identified. This study aimed to identify metabolism-related differentially expressed genes as potential biomarkers for pathogenesis of ITP using bioinformatic analyses.The microarray expression data of the peripheral blood mononuclear cells were downloaded from the Gene Expression Omnibus database (GSE112278 download link: https://www.ncbi.nlm.nih.gov/geo/query/acc.cgi?acc=GSE112278). Key module genes were intersected with metabolism-related genes to obtain the metabolism-related key candidate genes. The hub genes were screened based on the degree function in the coytoscape sofware. The key ITP-related genes were subjected to functional enrichment analysis. Immune infiltration analysis was performed using a single-sample gene set enrichment analysis algorithm to evaluate the differential infiltration levels of immune cell types between ITP patient and control. Molecular subtypes were identified based on the expression of hub genes. The expression of hub genes in the ITP patients was validated using quantitative real-time polymerase chain reaction analysis. This study identified five hub genes (*ADH4*, *CYP7A1*, *CYP1A2*, *CYP8B1*, and *NR1H4*), which were be associated with the pathogenesis of ITP, and two molecular subtypes of ITP. Among these hub genes, CYP7A1 and CYP8B1 involved in cholesterol metabolism,were further verified in clinical samples.

## Introduction

Immune thrombocytopenia (ITP), an acquired autoimmune hematological disease, is characterized by increased platelet destruction and decreased platelet production^1^. The annual incidence of ITP is estimated to be 3–4 cases per 100,000 individuals. The incidence of ITP, which is slightly more common in females than males, is the highest in children and patients aged > 60 years^2^. Clinically, ITP-related bleeding symptoms widely vary, ranging from bleeding in the skin and mucosal regions to severe visceral hemorrhage, and can be life-threatening. The mortality rate in adults with ITP is 1.3–2.2 times higher than that in the general population^3^. The complex pathogenesis of ITP has not been completely elucidated. Dysfunctional proliferation of autoreactive T cells is suggested to be the etiological factor for the loss of tolerance to platelet autoantigens in ITP^4^. Additionally, previous studies have demonstrated that B lymphocytes and natural killer (NK) cells are involved in the pathogenesis of ITP^5^. Thus, the major pathogenetic mechanism of ITP involves the loss of immune tolerance to platelet autoantigen, resulting in the aberrant activation of humoral and cellular immunity, the upregulation of platelet destruction, and the downregulation of platelet production by megakaryocytes^6^.

ITP is primarily diagnosed by excluding other causes of thrombocytopenia owing to the lack of unambiguous diagnostic markers. Thus, some patients with ITP can be misdiagnosed. One study reported that one in seven patients with suspected primary ITP was misdiagnosed at some point during the disease course^7^. Hence, in clinical practice, suspected patients are diagnosed based on medical history, physical examination, peripheral blood cell count, and peripheral blood smear. Splenomegaly and concomitant symptoms, which are aberrant clinical presentations, should be examined carefully to exclude the presence of other underlying diseases^8^. To decrease the rate of misdiagnosis, bone marrow examination and antinuclear antibody, anti-phospholipid antibody, anti-thyroid antibody, thyroid function, and coagulation parameter tests were recommended in the recent Chinese ITP guideline^9^. The diagnosis of ITP may be further complicated in clinical practice. Approximately 60–70% of ITP cases are estimated to progress to persistent or chronic ITP^10^. The annual incidence of refractory ITP is 100 cases per million individuals^11^.

Alterations in the metabolome are implicated in disease development^12^. A case–control study of metabolomics illustrated that ITP was related to phenylalanine, tyrosine, and tryptophan biosynthesis-related, phenylalanine metabolism-related, and glyoxylate and dicarboxylate metabolism-related^13^. However, the correlation between these metabolic alterations and metabolism-related genes in the occurrence of ITP has not been elucidated, preventing the clinical application of these potential biomarkers. Thus, there is a need to identify potential biomarkers for pathogenesis of ITP to improve diagnostic accuracy and to guide treatment decisions.

Metabolic alterations are associated with the pathogenesis of several diseases, including cancer, diabetes, metabolic disorders, and neurodegeneration. The differential serum metabolite levels between patients with acute leukemia and healthy controls indicated a shift in energy metabolism. This study aimed to identify metabolism-related hub genes involved in the pathogenesis of ITP using public datasets. The expression data were obtained from the Gene Expression Omnibus (GEO) database (GSE112278) and subjected to integration analysis. Comprehensive bioinformatics and enrichment analyses were performed to identify the differentially expressed genes (DEGs) and their functions in ITP. Key module genes were screened using weighted gene co-expression network analysis (WGCNA) and intersected with metabolism-related genes to obtain the metabolism-related key candidate genes. Furthermore, a protein–protein interaction (PPI) network was constructed using the STRING database and the Cytoscape program to screen hub genes. The following five hub genes were identified: *ADH4*, *CYP7A1*, *CYP1A2*, *CYP8B1*, and *NR1H4*. The expression levels of these five hub genes in clinical peripheral blood samples were validated using quantitative real-time polymerase chain reaction (qRT-PCR). The potential diagnostic values of CYP7A1 and CYP8B1 for ITP were examined using the receiver operating characteristic (ROC) curve. The hub genes identified in this study can may provide novel insights into the mechanisms underlying ITP pathogenesis and serve as potential diagnostic biomarkers for ITP.

## Methods

### Data source

One study reported that clonal T-cell correlates of response and non-response to eltrombopag therapy according to blood transcriptome analysis in a cohort of patients with chronic immune thrombocytopenia^14^. The gene expression datasets GSE112278 and GSE183073 were downloaded from the GEO database (http://www.ncbi.nlm.nih.gov/geo), which is an open- source repository of next-generation sequencing data, hybridization arrays, chips, and microarrays^15^. The download datasets were merged by combat method using R sva package. The merged dataset comprised the sequencing data of peripheral blood samples of 17 patients with ITP and 7 healthy control subjects (ITP patient dataset download link: https://www.ncbi.nlm.nih.gov/geo/query/acc.cgi?acc=GSE112278 and healthy control dataset download link: https://www.ncbi.nlm.nih.gov/geo/query/acc.cgi?acc=GSE183073).

### Identification of DEGs in patients with ITP

The DEGs between the ITP and control groups were identified using the R package “limma” based on the following criteria: |log2 fold-change (FC)|> 0.5; P < 0.05. The heatmap was generated using the R packages “pheatmap” and “dplyr.” The top 25 significantly upregulated and downregulated genes were used for constructing the differential gene heatmap.

### Screening of key modules and target genes based on WGCNA

WGCNA was performed to identify a specific clinical features-related gene set. The WGCNA method was adopted to gene expression data using the “WGCNA”R package to identify the correlation between gene expression and the ITP-related disease phenotype. Outlier samples were examined by using hierarchical clustering, then followed by scale-free network construction. An adjacent matrix was constructed by adopting the optimal soft threshold power(β = 5, R^2^ = 0.95), which was gained from the pick soft function analysis and transformed into a corresponding topological overlap matrix (TOM). The gene network was hierarchically clustered adopting l—TOM as the distance measure to screen the gene groups (module eigengenes, ME) whose expression varied across clinical features. Modules were merged if the correlations of their ME exceeded a threshold(0.75). Pearson correlation analysis was adopted to uncover the correlations between modules and clinical features.

### PPI construction and hub gene screening

The PPI network map of the candidate genes was mapped to the STRING database (https://string-db.org) assembly and visualized using the “Cytoscape” software^16^. Next, the STRING database was used for interaction analysis of candidate genes. As PPI analysis can aid in the identification of hub genes with core functions, the PPI of genes in the identified key modules was further examined^17^. Cytoscape software was used to identify the important nodes in the network^16^ and the hub genes from the whole PPI network using the degree function. According to degree, the top 5 genes are screened out as the hub genes.

### Enrichment analysis

Gene Ontology (GO) enrichment and Genes and Genomes (KEGG) pathway analyses are performed with the packages “clusterProfiler”, “org.Hs.eg.db”, and “ggplot2” and a cut-off criterion of a P value < 0.05. DEGs were subjected to GO enrichment analysis, which is an informatic method to identify the significant enrichment of biological functions in the GO terms biological process (BP), cellular component (CC), and molecular function (MF)^18^. Additionally, DEGs were subjected to KEGGpathway enrichment analysis using the KEGG database^19^.

### Gene set enrichment analysis (GSEA)

GSEA is a gene set-based algorithm that is used to construct a database of molecular characteristics according to known information, including gene characteristics, location, and biological functions^20^. Single-sample GSEA (ssGSEA) was performed using the GSVA R package. The scores of the relevant immune pathways were calculated based on the gene expression matrix of individual samples using the ssGSEA method^21^. The “vioplot” package was used to calculate the scores between the ITP and control groups and compare the activity of immune-related pathways. The results were visualized using the pheatmap R package. The Pearson correlation between the expression levels of hub genes and the scores of the immune-related pathway activities in the ITP group were compared using the R package “ggplot2” and “reshape2,” respectively.

The scores of the proportion of immune cells based on the gene expression matrix were determined using ssGSEA with the GSVA R package. The “vioplot” package was used to calculate the scores between the ITP and control groups to compare the proportion of cells in different immune-related pathways, and the results were visualized by heatmap. The Pearson correlation between the expression levels of hub genes and the relative proportions of immune cells in the ITP group were compared using the R packages “ggplot2” and “reshape2,” respectively.

The GSVA R package was used to perform ssGSEA.

The background reference geneset of metabolism-related pathways was from the a previous study^22^ and the metabolism-related pathways are downloaded from the official website (https://www.gsea-msigdb.org/gsea/index.jsp;). The ssGSEA method was used to calculate the enrichment scores of different metabolism-related pathways between the ITP and control groups based on the gene expression matrix for each sample. The differential metabolic pathways between the ITP and control groups were screened using the “limma” package in R, and the results were visualized using a heatmap.The Pearson correlation between the expression levels of hub genes in the ITP group and the relative proportions of enrichment in differential metabolic pathways between the ITP and control groups were compared using the R packages “ggplot2” and “reshape2,” respectively.

### Identification of ITP molecular subtypes

As ITP exhibits heterogeneity, the presence of distinct ITP subtypes was determined. The concordant clustering algorithm was used to cluster molecular subtypes according to the expression level of hub genes in ITP samples using the Consensus Cluster Plus R package. The optimal cluster number k was determined according to the cumulative distribution function (CDF) and area under the CDF curve^23^. In this study, the number of clusters of subgroups was determined according to the CDF value of > 0.8^24^. The R package limma was used to compare the differential expression of hub genes in different subtypes. Principal component analysis (PCA) is a multidimensionality-reduction technique used to visualize similarities and differences between samples^25^. The PCA result was visualized using the ggplot2 package. Cell-type identification by estimating relative subsets of RNA transcripts (CIBERSORT) analysis was performed to calculate the proportion of 22 infiltrating immune cell types in different ITP molecular subtypes according to the gene expression signature. The predicted results were filtered based on the criterion P < 0.05. The GSVA R package was used to perform ssGSEA. The KEGG signaling and metabolic pathways were compared among the subtypes. The KEGG gene set “c2.cp.kegg.symbols.gmt” was used to retrieve various metabolism-related pathways from the literature to screen differential signaling pathways and differential metabolism-related pathways between the two subtypes.

### Hub gene validation

The expression levels of hub genes in the peripheral blood samples of patients with ITP and healthy controls were determined using qRT-PCR analysis. The samples of 39 patients with ITP and 21 healthy controls were collected from the First Affiliated Hospital of Chongqing Medical University, Chongqing, China, the Affiliated Hospital, Southwest Medical University and the Affiliated Traditional Chinese Medicine Hospital, Southwest Medical University, Luzhou, China. The clinical characteristics of 39 patients with ITP are shown in Table 1. Total RNA was isolated from the peripheral blood sample using an adsorption column (Mei5 Biotechnology Co. Ltd) and reverse-transcribed into complementary DNA using the PrimeScript™ RT reagent kit with a gDNA eraser (Mei5 Biotechnology Co. Ltd). The optical density value was measured to calculate the concentration and purity of RNA. qRT-PCR analysis was performed using the M5 One-Step q-PCR kit (SYBR green) (Mei5 Biotechnology Co. Ltd) with an Applied Scan Fast Real-Time PCR System with Step One Plus Real-Time. All procedures were performed following the manufacturer’s instructions. The expression levels of hub genes were normalized to those of *GAPDH*. The relative expression level was calculated using the 2^−ΔΔCt^ method. The primers used in this study are listed in Supplementary Material Table 1. The amplification was performed using a two-step PCR protocol under the following conditions: 95 °C for 30 s, followed by 40 cycles of 95 °C for 5 s and 60 °C for 30 s.

**Table 1:** Clinical characteristics of patients with immune thrombocytopenia (ITP).

Baseline characteristics	ITP statistics
Age, median (range) in years	54 (16–84)
Sex, n (%)	
Female	23 (58.97)
Male	16 (41.03)
Type of ITP, n (%)	
New diagnosis (duration: < 3 months)	18 (46.2)
Persistent (duration: 3–12 months)	2 (5.1)
Chronic (duration: > 12 months)	19 (48.7)
Baseline platelet count, median (range)	8 (0–56) × 10^9^
Prior treatment, n (%)	
None	4 (10.3)
Corticosteroids	31 (79.5)
Immunoglobulins	6 (15.4)
TPO-RA agonists	0
Rituximab	0
Splenectomy	0

*TPO-RA* thrombopoietin receptor agonists.

### Statistical analysis

All statistical analyses were performed using R software (version 3.6.0) and GraphPad Prism 9. Means between the groups were compared using the unpaired t-test. Differences were considered significant at P < 0.05.

### Ethics approval and consent to participate

This study was carried out in accordance with the Helsinki declaration and approved by the ethics committee of The First Affiliated Hospital of Chongqing Medical University.

## Results

### Identification of DEGs

**Figure 1:**
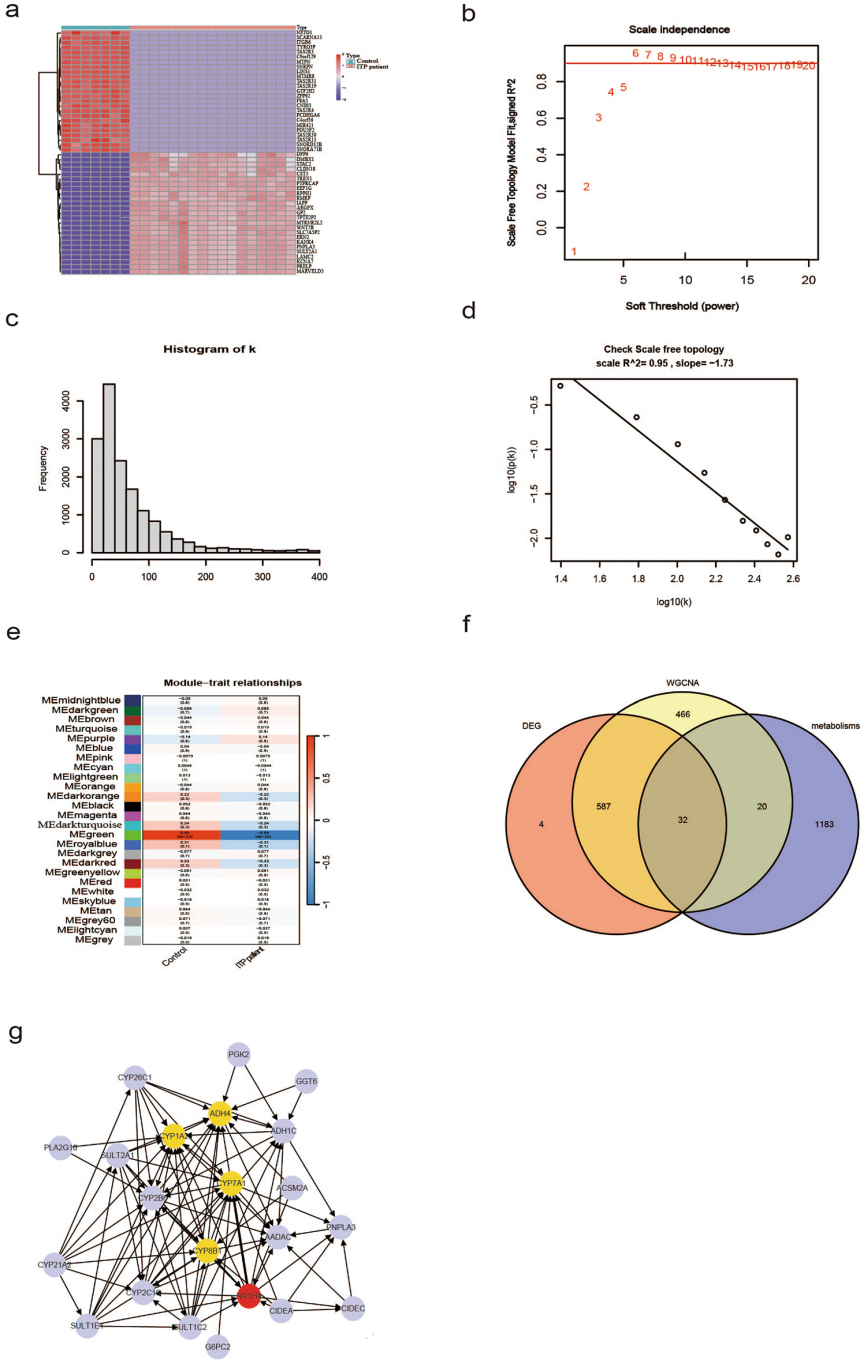
(**a**) Heat map of the DEGs between ITP patient and control; (**b**) The values of soft-threshold power based on scale independence and mean connectivity; (**c**) and (**d**) Check scale-free topology. The correlation coefficient of the connection degree k and p(k) was 0.95, indicating scale-free topology was constructed; (**e**) Heatmap of the module-trait relationships; (**f**) The result of Venn diagram of the intersection genes via DEGs WGCNA analysis, and GSEA datdbase; (**g**) The results of protein–protein interaction network.

DEGs were identified in the single-cell RNA sequencing dataset GSE112278 based on the following criteria: P < 0.05; |log2FC|> 0.5. The top 25 upregulated and the top 25 downregulated genes were selected to construct a heatmap (Fig. 1a).

### WGCNA and identification of the key module and hub genes

The key parameter associated with a scale-free network is the soft threshold power value. In this study, the soft threshold power value for screening the gene set associated with the clinical phenotype of ITP was 5 to achieve scale independence of 0.95 (power = 5) (Fig. 1b–d). The MEgreen module was correlated with ITP ((r = 0:99, P = 4e − 23)) and was selected for further analysis (Fig. 1e). In total, 1341 metabolism-related genes were retrieved from the GSEA database. The Venn diagram of DEGs, WGCNA-derived genes, and metabolism-related gene sets revealed 32 intersection genes (Fig. 1f). A PPI network was constructed using these candidate genes. Based on the degree score, five hub genes were identified from the PPI network (Fig. 1g).

### GO and KEGG pathway analyses

**Figure 2:**
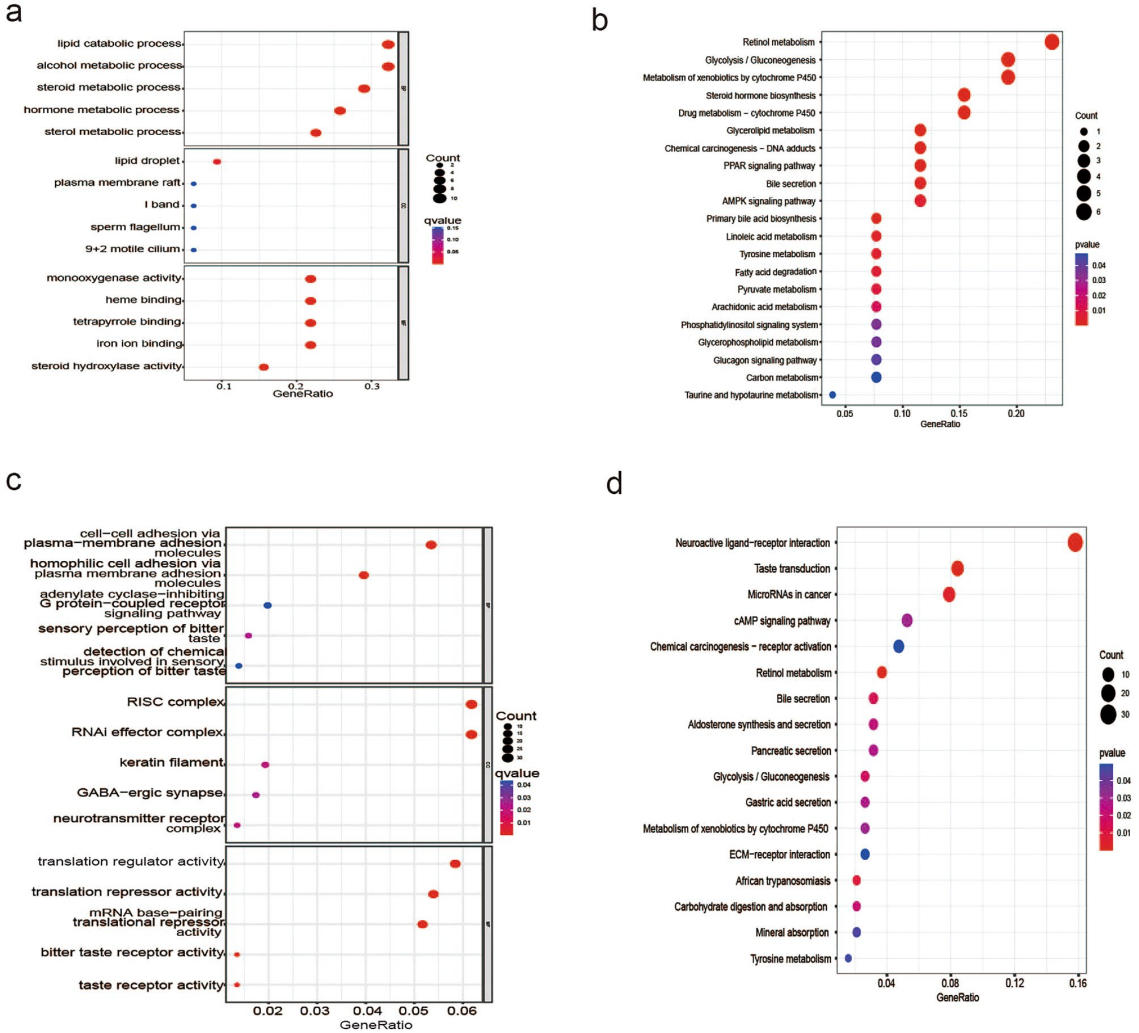
Functional enrichment analysis of intersection genes and DEGs. Intersection genes were obtained from DEGs, WGCNA analysis and metabolism-related genes. (**a**) The results of GO analysis of intersection genes; (**b**) The results of KEGG analysis of intersection genes; (**c**) The results of GO analysis of DEGs; (**d**) The results of KEGG analysis of DEGs. *GO* gene ontology, *KEGG* Kyoto Encyclopedia of Genes and Genome.

GO enrichment analysis revealed that the intersection genes obtained from DEGs, WGCNA analysis and metabolism-related genes were enriched in various terms as follows: BP term: lipid catabolic process, steroid metabolic process, and hormone metabolic process; CC term: lipid droplet; MF term: monooxygenase activity and steroid hydroxylase activity (Fig. 2a). KEGG enrichment analysis revealed that intersection genes were enriched in the following pathways: retinol metabolism, glycerolipid metabolism, primary bile acid biosynthesis, bile secretion, tyrosine metabolism, fatty acid degradation, arachidonic acid metabolism, phosphatidylinositol signaling system, AMPK signaling pathway, PPAR signaling pathway, and glucagon signaling pathway (Fig. 2b).

Additionally, GO enrichment analysis revealed that the DEGs were enriched in various terms as follows: BP term: cell–cell adhesion via plasma membrane adhesion molecules, homophilic cell adhesion via plasma membrane adhesion molecules, sensory perception of bitter taste, adenylate cyclase-inhibiting G protein-coupled receptor signaling pathway, and detection of chemical stimulus involved in sensory perception of bitter taste; CC term: RISC complex, RNAi effector complex, keratin filament, and neurotransmitter receptor complex; MF term: mRNA base-pairing translational repressor activity, mRNA base-pairing translational repressor activity, bitter taste receptor activity, and taste receptor activity (Fig. 2c). KEGG enrichment analysis revealed that the DEGs were enriched in the following pathways: taste transduction, neuroactive ligand-receptor interaction, retinol metabolism, microRNAs in cancer, bile secretion, pancreatic secretion, cAMP signaling pathway, tyrosine metabolism, and metabolism of xenobiotics by cytochrome P450 (Fig. 2d).

### ssGSEA

**Figure 3:**
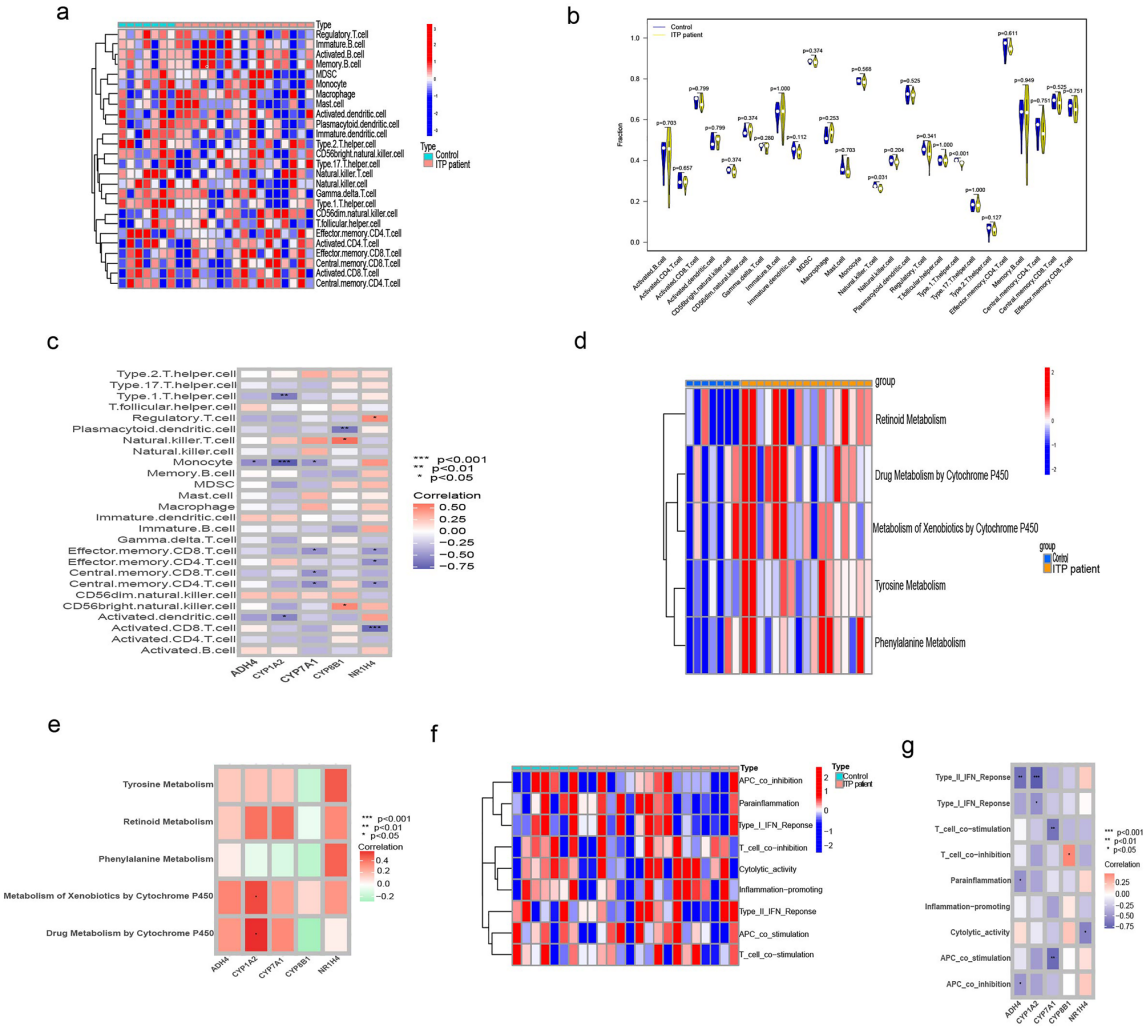
(**a**–**c**) The result of GSVA analysis; (**d**) differential metabolism-related pathways between ITP patient and control; (**e**) the correlation analysis results of the hub genes and metabolism-related pathways; (**f**) differential immune-related pathways between ITP patient and control; (**g**) the correlation analysis results of the hub genes and immune-related pathways. *ITP* Immune thrombocytopenia.

ssGSEA was performed to further compare the scores of immune cells and immune-related pathways between the ITP and control groups. The GSE112278 dataset was subjected to ssGSEA to examine the relative infiltration abundance of 26 immune cell subpopulations in the ITP and healthy control groups, and the results were represented as a heatmap (Fig. 3a). The violin plot of immune cell infiltration revealed that compared with those in the healthy control group, the infiltration levels of Th1 cells and NK cells were upregulated in the ITP group (Fig. 3b). In this study, ssGSEA revealed that the five hub genes were strongly correlated with immune cells. *ADH4* expression was negatively correlated with monocyte abundance. *CYP1A2* expression was negatively correlated with activated dendritic cell, monocyte, and Th1 cell abundances. *CYP7A1* was negatively correlated with central memory CD4 + T cell, central memory CD8 + T cell, effector memory CD8 + T cell, and monocyte abundances. *CYP8B1* expression was positively correlated with CD56 bright NK cell and NK T (NKT) cell abundances but was negatively correlated with plasmacytoid dendritic cell abundance. *NR1H4* was negatively correlated with activated CD8 T cell, central memory CD4 T cell, effector memory CD4 T cell, and effector memory CD8 T cell abundances and positively correlated with regulatory T cell abundance (Fig. 3c).

### Pathway analysis

The heatmap of differential metabolism-related pathways between the ITP and control groups revealed the enrichment of retinoid metabolism, drug metabolism by cytochrome P450, metabolism of xenobiotics by cytochrome P450, tyrosine metabolism, and phenylalanine metabolism (Fig. 3d). Correlation analysis of the hub genes and metabolism-related pathways demonstrated that *CYP1A2* was significantly and positively correlated with the metabolism of xenobiotics by cytochrome P450 and drug metabolism by cytochrome P450(P < 0.05, Fig. 3e).

The heatmap of differential immune-related pathways between the ITP and control groups revealed the enrichment of antigen-presenting cell (APC) co-inhibition, parainflammation, type I IFN response, T cell co-inhibition, cytolytic activity, inflammation-promoting activity, type II IFN response, APC co-stimulation, and T cell co-stimulation (Fig. 3f). The correlation analysis of the hub genes and immune-related pathways demonstrated that *ADH4* expression was negatively correlated with APC co-inhibition and parainflammation. *CYP1A2* expression was negatively correlated with type I IFN response and type II IFN response. *CYP7A1* expression was negatively correlated with APC co-stimulation and T cell co-stimulation. *CYP8B1* expression was positively correlated with T cell co-inhibition. *NR1H4* expression was negatively correlated with cytolytic activity (Fig. 3g).

### Molecular subtypes of ITP samples

**Figure 4:**
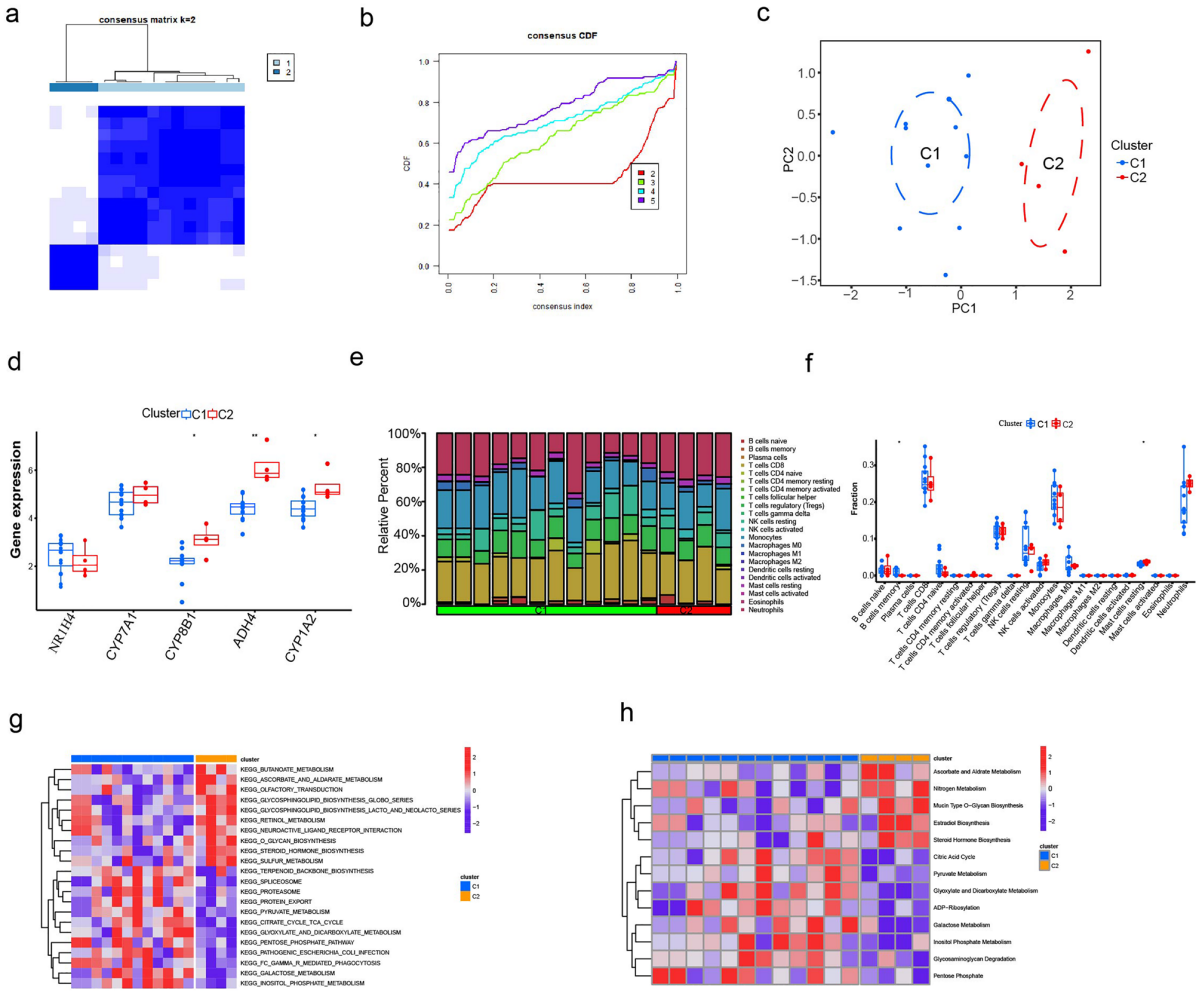
(**a**) and (**b**) Molecular subtype analysis in ITP; (**c**) PCA analysis; (**d**) the expression of hub genes in molecular subtypes; (**e**) and (**f**) the relative percent of immune cells infiltration between the two subtypes; (**g**) the heat-map of KEGG pathway between the C1 and C2 subgroups; (**h**) the heat-map of metabolism-related pathway between the C1 and C2 subgroups. *ITP* Immune thrombocytopenia, *PCA* principal component analysis, *KEGG* Kyoto Encyclopedia of Genes and Genome.

To further explore the profile and characteristics of five metabolism-related hub genes in ITP, a consensus clustering algorithm was used to stratify patients with ITP based on the expression levels of five hub genes. The consistency coefficient was calculated to obtain the optimal clustering number (k value). This study determined that k = 2 was the optimal clustering number for stratifying the entire cohort into cluster 1 (C1) and cluster 2 (C2) (Fig. 4a,b). PCA revealed that patients with ITP were distinctly clustered into two clusters (Fig. 4c). The expression levels of the hub genes in the two molecular subtypes are shown in Fig. 4d. The *CYP8B1*, *ADH4*, and *CYP1A2* expression levels in the C2 subtype were significantly upregulated when compared with those in the C1 subtype. However, the *NR1H4* and *CYP7A1* expression levels were not significantly different between the C1 and C2 subtypes. The differential infiltration levels of immune cells between the two molecular subtypes were examined. The infiltration of B cells memory and mast cells resting significantly varied between the two subtypes (Fig. 4e,f). Functional pathway enrichment analysis using the GSVA algorithm revealed that 22 KEGG signaling pathways and 13 metabolism-related pathways significantly varied between the C1 and C2 subtypes (Fig. 4g,h).

### Validation of hub genes

**Figure 5:**
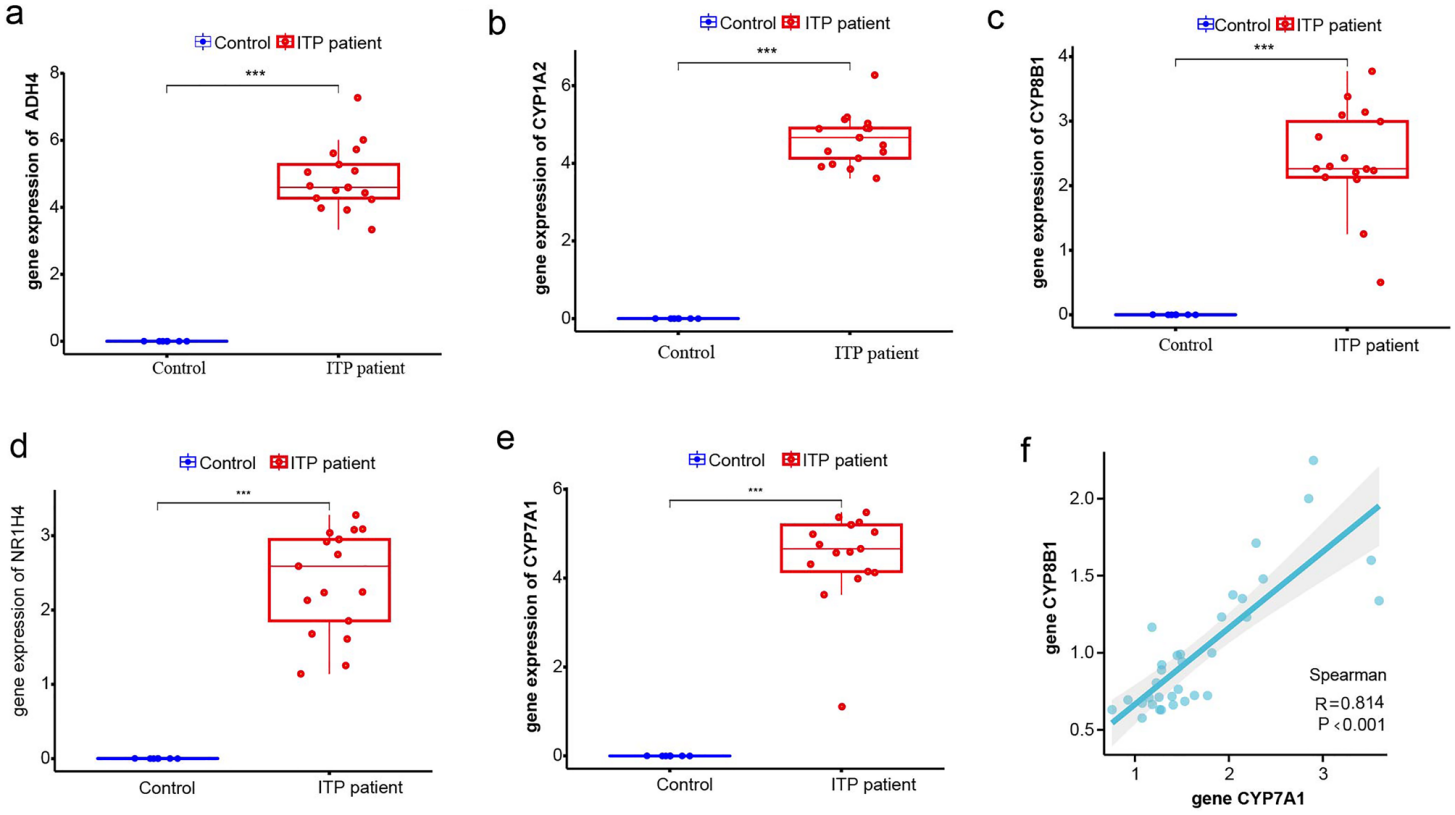
(**a**–**e**) *ADH4*, *CYP1A2*, *CYP8B1*, *NR1H4*, and *CYP7A1* expression between ITP patient and control; (**f**) the correlation of *CYP8B1* and *CYP7A1* expression. *ITP* Immune thrombocytopenia.

**Figure 6:**
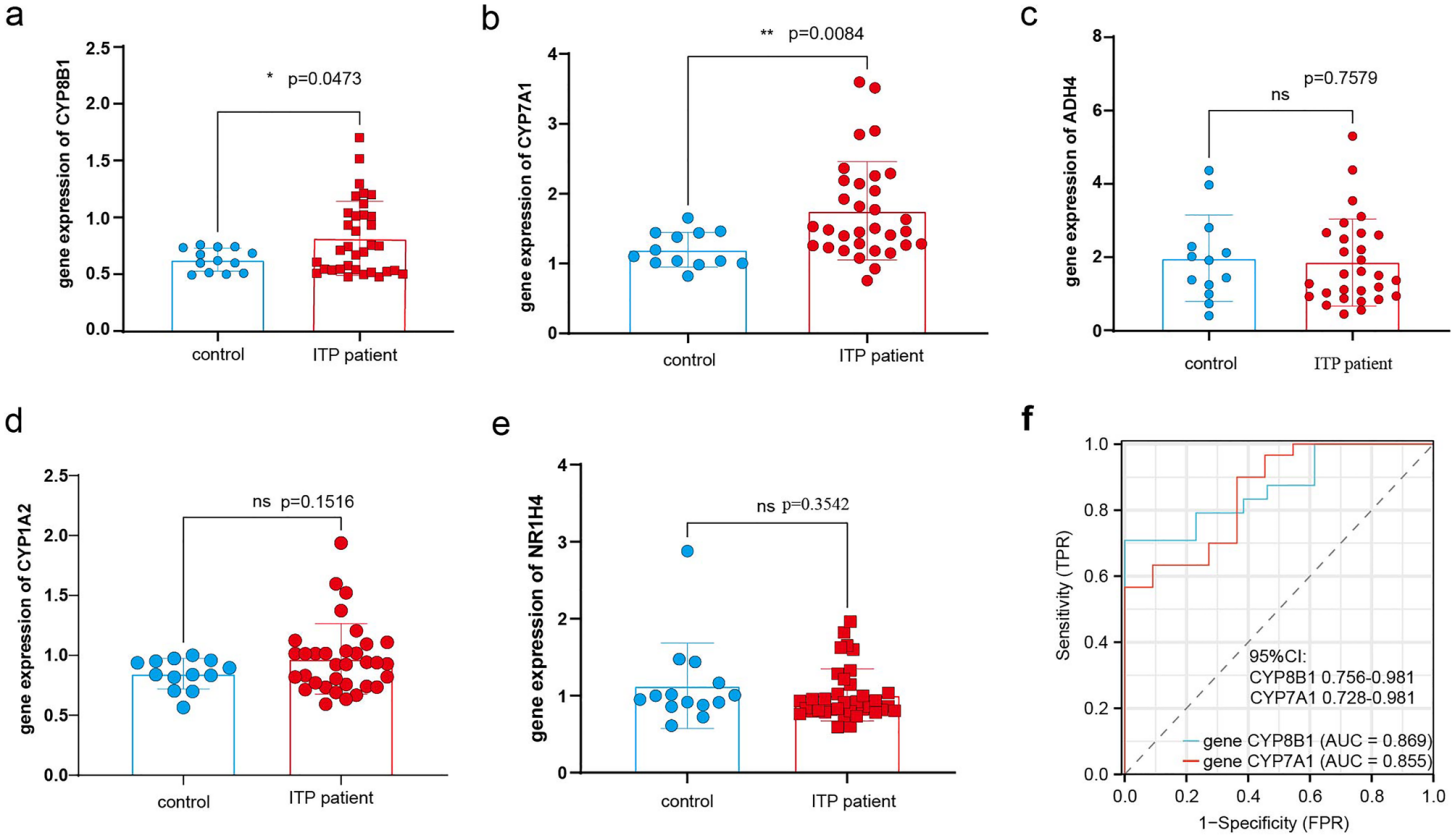
(**a**–**e**) the verifited *CYP8B1*, *CYP7A1*, *ADH4*, *CYP1A2*, and *NR1H4* expression between ITP patient and control; (**f**) ROC analysis of the *CYP7A1* and *CYP8B1*. *ITP* Immune thrombocytopenia, *ROC* receiving operating characteristic.

This study examined the expression levels of five ITP pathogenesis-related hub genes (*CYP7A1*, *NR1H4*, *CYP8B1*, *CYP1A2*, and *ADH4*). The expression levels of these genes were upregulated in the ITP group (Fig. 5a–e). qRT-PCR analysis of clinical samples revealed that compared with those in the healthy control group, the expression levels of *CYP8B1* and *CYP7A1* were significantly upregulated in the ITP group (Fig. 6a,b),however, the *ADH4*, *CYP1A2*, and *NR1H4* genes were not differentially expressed (Fig. 6c–e). Additionally, the expression levels of *CYP7A1* were positively correlated with those of *CYP8B1* (Fig. 5f). Additionally, the diagnostic potential of the five ITP-related hub genes was examined using the ROC curves. The area under the curve values of *CYP8B1* and *CYP7A1* were 0.869 (95% confidence interval (CI): 0.756–0.981) and 0.885 (95% CI: 0.728–0.981), respectively. ROC curve verification revealed that the specificity and sensitivity of *CYP8B1* and *CYP7A1* were high for diagnosing ITP (Fig. 6f).

## Discussion

ITP is an autoimmune disease, which is mainly characterized by humoral and cellular immune-mediated platelet destruction and impaired platelet production^26^. However, the pathogenesis and etiology of ITP remain not fully understood and the “gold standard” diagnostic criteria are lacking. Previous metabolomics studies have found differences in metabolic characteristics between the healthy and ITP patients^13^ and provided novel insights into the pathogenic mechanisms of ITP involving gut microbiota, cytokine, and fatty metabolism. These findings can improve differential diagnosis and support treatment decisions for patients with ITP^27^. But the profiles of genes related to ITP metabolism remains uncertain. This study performed bioinformatics analysis to identify the DEGs and five metabolism-related central genes associated with ITP. The *CYP7A1* and *CYP8B1* expression levels were validated in patients with ITP. The dysregulation of *CYP7A1* and *CYP8B1* is involved in the pathogenesis of ITP. Thus, *CYP7A1* and *CYP8B1* are potential diagnostic biomarkers for ITP. However, three other biomarkers could not be validated in the clinical samples. This can be attributed to the differences in sample size, the characteristics of patients, or the heterogeneity of public expression datasets.

*CYP7A1* encodes cholesterol 7α-hydroxylase, which is the key enzyme of bile acid synthesis and initiates the classical pathway of bile acid synthesis^28,29^. In the study, *CYP7A1* was negatively correlated with T cell co-stimulation and APC co-stimulation. ssGSEA revealed that *CYP7A1* was negatively correlated with central memory CD4 + T cell, central memory CD8 T cell, and effector memory CD8 + T cell abundances. The interaction of B7 family molecules on the APC surface with CD28 family molecules on the T cell surface provides the second signal, which is known as the co-stimulation signal^30^. T cell activation, proliferation, and differentiation are dependent on the interaction between T cell co-stimulation molecules and their receptors on the APC surface^31^.

mTOR may act to integrate costimulatory signals, which in turn direct the outcome of T cell differentiation and activation^32^. The mTOR signaling pathway determines T cell fate, including the differentiation of naive cells into effector T cells or T regulatory (Treg) cells and the development of CD8 + memory T cells^33–37^. In ITP, bone marrow CD8 + T cells, which are reported to be platelet-specific, are activated, impairing the apoptosis of megakaryocytes and suppressing platelet production^38^. Sirolimus is a mammalian target of rapamycin (mTOR) inhibitor that has been demonstrated to inhibit lymphocyte activity and that demonstrated efficacy as a second-line agent for refractory/relapsed ITP^39^. This indicates that mTOR signaling pathway is involved in the pathogenesis of ITP. In this study, CYP7A1 expression in patients with ITP was higher than that in healthy subjects. Consistently, the expression of CYP7A1 was upregulated in the peripheral blood samples of patients with ITP. Therefore, it was speculated that CYP7A1 mediates the pathogenesis of ITP through the mTOR pathway.

*CYP8B1* encodes sterol 12α-hydroxylase, which is necessary for the synthesis of cholic acid^40^. In the study, CYP8B1 expression was positively correlated with CD56 bright NK and NKT cell abundances. CD56 bright NK cells represent distinct human NK cell subsets with differing physiological roles^41^. NKT cells share the properties of both T and NK cells^42–44^. ElRashedi et al. examined NK cells in pediatric patients with ITP and reported that childhood ITP is associated with the upregulation of cytotoxic T lymphocytes and the downregulation of peripheral blood NK cells although the reasons for these observations are unclear^45^. The activation of the MAPK pathway, especially ERK activation, promotes NK cell proliferation and development^46^. In this study, CYP8B1 expression in patients with ITP was higher than that in healthy subjects. Consistently, CYP8B1 expression was upregulated in the peripheral blood samples of patients with ITP. Thus, it was speculated that CYP8B1 mediates the pathogenesis of ITP through the MAPK pathway by affecting the development and proliferation of NK cells. Moreover, ROC curve verification demonstrated that *CYP7A1* and *CYP8B1* exhibited high specificity and sensitivity for predicting ITP.

*ADH4* encodes an alternative alcohol dehydrogenase, which plays an important role in metabolizing various substrates, including ethanol and retinol^47^. In this study, *ADH4* expression was negatively correlated with type II IFN response. IFN-γ is a cytokine mainly produced by activated NK cells, cytotoxic T cells and Th1 cells^48^, which plays a critical role in cellular immunity^49^. Study showed that interferon-γ is significant higher concentrations in ITP patients than that in healthy controls^50^. Activation of MAPK signaling pathway involves in T-cell and NK cell activation and proliferation proliferation^46^. In this study, ADH4 expression in patients with ITP was higher than that in healthy controls, but it wasn’t validated in the peripheral blood samples from ITP patients. ADH4 may play a protective role in the progression of ITP. Thus, it was speculated that ADH4 mediates the pathogenesis of ITP via the MAPK pathway.

CYP1A2 belongs to CYP450 superfamily^51^. The human CYP450 enzyme superfamily catalyzes the oxidative metabolism of various drugs, xenobiotics, and other endogenous substances^52^. In this study, CYP1A2 expression was positively correlated with the metabolism of xenobiotics by cytochrome P450. One study from Canada reported that CYP1A2 plays a role in the production of reactive oxygen species (ROS)^53^. ROS are products of oxidative metabolism^54^. Study revealed that the levels of ROS in chronic ITP were upregulated when compared with those in healthy volunteers^55^. In this study, CYP1A2 expression in patients with ITP was higher than that in healthy controls, but it wasn’t validated in the peripheral blood samples from ITP patients.Thus, it was speculated that CYP1A2 mediates the pathogenesis of ITP via promoting the generation of ROS.

*NR1H4* (also called FXR) encodes ligand-activated transcription factors^56^. Immune cell infiltration analysis revealed that NR1H4 expression was positively correlated with Treg cell abundance. Treg cells inhibit T cell-mediated immunity and are involved in immunological tolerance^57^. Treg cell deficiency has been associated with the pathogenesis of ITP^58^. Treg differentiative activity is mediated by the PIK3/AKT signaling pathway^59^ and Treg is tightly controlled by mTORC1 activation^60^. NR1H4 might reduce cholesterol biosynthesis by inhibiting the PI3K/AKT/mTOR signaling pathway^61^ and cholesterol is essential for lymphocyte activation^62^. This demonstrated that PI3K/Akt/mTOR signaling pathway plays an important role in Treg differentiation and activity. In this study, NRIH4 expression in patients with ITP was higher than that in healthy controls, but it wasn’t validated in the peripheral blood samples from ITP patients. Thus, it was speculated that NR1H4 mediates the pathogenesis of ITP via PI3K/Akt/mTOR signaling pathway.

ITP is a highly heterogeneous disease. Previous studies have demonstrated that the clinical manifestation, clinical consequences, and treatment responses of ITP markedly vary^63^. The hemorrhagic symptoms vary from an asymptomatic state to a life-threatening hemorrhage. Some patients fail to respond to glucocorticoid (GC) therapy. ITP has not been previously classified based on the expression levels of metabolism-related genes. This study suggested that metabolism-related genes are involved in the pathogenesis of ITP and ITP was classified into two molecular subtypes based on the expression of the five ITP-related biomarkers.

Atorvastatin lowers cholesterol through inhibiting the HMG-CoA reductase of the mevalonate pathway for cholesterol biosynthesis. It has been reported that atorvastatin may have a potential therapeutic effect in treating ITP^64^ and this showed that cholesterol biosynthesis was increased in ITP patients. Cholesterol is a precursor of steroid hormones, oxysterols, and bile acids^65^. Steroid hormones are classified into the following five groups: GCs, mineralocorticoids, androgens, estrogens, and progestogens^66^. In the study, *CYP8B1 was* significantly upregulated in the C2 subtype, involving in the conversion of cholesterol to cholic acid. Pathway analysis revealed that steroid hormone biosynthesis in the C2 subtype was upregulated when compared with that in the C1 subtype. Meanwhile, the citric acid cycle in the C1 subtype was upregulated when compared with that in the C2 subtype. However, GCs exert contrasting effects on macrophages depending on the level and time of exposure. The upregulation of GC exerts anti-inflammatory and immunosuppressive effects, while the downregulation of GC facilitates macrophage polarization into pro-inflammatory phenotypes^66^. The citric acid cycle, a key cellular metabolic pathway, provides energy for cellular metabolism^67^. The metabolites of the citric acid cycle are involved in the regulation of immune responses^68^. Remodeling of the citric acid cycle is a metabolic adaptation associated with inflammatory macrophage activation^69^. The proportion of anti-inflammatory macrophages is downregulated in the mouse ITP model, as well as in patients with ITP^70,71^. The results suggest that the different levels of inflammation between the two subtypes. Thus, it was speculated that the response of patients with the C1 subtype to anti-inflammation treatment may be higher than that of patients with the C2 subtype.

CIBERSORT analysis revealed that the infiltration levels of immune cell types significantly varied between the two subgroups. The abundances of B cells memory and mast cells were significantly upregulated in the C1 and C2 subtypes, respectively. Memory B cells are reported to be resistant to rituximab^72^. The mast cells can produce IL-17^73^. The treatment of ITP associated with IL-17-mediated macrophages is challenging^74^. Thus, it was speculated that different molecular subtypes exhibit different responses to the same therapy.

## Conclusions

*ADH4*, *CYP7A1*, *CYP1A2*, *CYP8B1*, and *NR1H4* are involved in pathogenesis of ITP. Additionally, *CYP8B1* and *CYP7A1* were identified as potential novel diagnostic biomarkers for ITP. The molecular subtypes may allow us to explore and understand the heterogeneity of ITP.

## Supplementary Information

Supplementary Table 1.

## Data Availability

All data in this study are available by contacting corresponding authors.
