# Author Correction: Identification of metabolism‑related key genes as potential biomarkers for pathogenesis of immune thrombocytopenia

**DOI:** 10.1038/s41598-025-08742-4

**Published:** 2025-07-17

**Authors:** Xiangmei Xu, Jiamin Zhang, Hongyun Xing, Liying Han, Xiaoming Li, Pengqiang Wu, Jirui Tang, Li Jing, Jie Luo, Jing Luo, Lin Liu

**Affiliations:** 1 Department of Hematology, The First Affiliated Hospital of Chongqing Medical University, No. 1 Youyi Road, Yuzhong District, Chongqing, 400016 People’s Republic of China; 2 Department of Oncology and Hematology, The Affiliated Traditional Chinese Medicine Hospital, Southwest Medical University, Luzhou, China; 3 Department of Hematology, The Affiliated Hospital, Southwest Medical University, Luzhou, China

Correction to: *Scientific Reports* 10.1038/s41598-024-59493-7, published online 19 April 2024.

The sub-section section “Date source” in the Methods section of the original version of this Article was incomplete.

“One study reported that clonal T-cell correlates of response and non-response to eltrombopag therapy according to blood transcriptome analysis in a cohort of patients with chronic immune^14^. The gene expression dataset GSE112278 was downloaded from the GEO database (http://www.ncbi.nlm.nih.gov/geo), which is an open-source repository of next-generation sequencing data, hybridization arrays, chips, and microarrays^15^. The GSE112278 dataset comprised the sequencing data of peripheral blood samples of 17 patients with ITP and 7 healthy control subjects (download link: https://www.ncbi.nlm.nih.gov/geo/query/acc.cgi?acc=GSE112278).”

now reads:

“One study reported that clonal T-cell correlates of response and non-response to eltrombopag therapy according to blood transcriptome analysis in a cohort of patients with chronic immune thrombocytopenia^14^. The gene expression datasets GSE112278 and GSE183073 were downloaded from the GEO database (http://www.ncbi.nlm.nih.gov/geo), which is an open- source repository of next-generation sequencing data, hybridization arrays, chips, and microarrays^15^. The download datasets were merged by combat method using R sva package. The merged dataset comprised the sequencing data of peripheral blood samples of 17 patients with ITP and 7 healthy control subjects (ITP patient dataset download link: https://www.ncbi.nlm.nih.gov/geo/query/acc.cgi?acc=GSE112278 and healthy control dataset download link: https://www.ncbi.nlm.nih.gov/geo/query/acc.cgi?acc=GSE183073). "

The original Article has been corrected.

