# Supplementary material for: Identification of metabolism-related key genes as potential biomarkers for pathogenesis of immune thrombocytopenia
Source: Sci Rep. 2024 Apr 19;14:9040. doi: 10.1038/s41598-024-59493-7 (PMC11031595; doi:10.1038/s41598-024-59493-7)
Supplement: Supplementary file 1 — Supplementary Table 1. [file 41598_2024_59493_MOESM1_ESM.docx]

**Supplementary Materials**

Supplementary Material Table 1: List of primers used in this study

| **Gene** | **Sequence** |
| --- | --- |
| ***GAPDH*^79^** | **F: 5′-CTCCTCCTGTTCGACAGTCAGC-3′** |
|  | **R: 5′-CCCAATACGACCAAATCCGTT-3′** |
| ***ADH4*^79^** | **F: 5′-GAAACCATGAAAGCAGCCCT-3′** |
|  | **R: 5′-CCAACCACCAAAGAATGTTCC-3′** |
| ***CYP7A1*^80^** | **F: 5′-CACAATGCCCGGGAGAAA-3′** |
|  | **R: 5′-AAAGTCGCTGGAATGGTGTTTG-3′** |
| ***CYP8B1*^81^** | **F: 5′-GGCAGGAGAGTTATTCATGGAG-3′** |
|  | **R: 5′-TTGTGAAAGAGACGCTGGAG-3′** |
| ***CYP1A2*^82^** | **F: 5′-GCCATTAACAAGCCCTTGAG-3′** |
|  | **R: 5′-ATGGCCAGGAAGAGGAAGAT-3′** |
| ***NR1H4*^83^** | **F: 5′-GCTTTGCTGAAAGGGTCTGC-3′** |
|  | **R: 5′-CAGAATGCCCAGACGGAAGT-3′** |
